# Macrophage makeover extreme viral edition: mechanisms of immune subversion and therapeutic perspectives

**DOI:** 10.1099/jgv.0.002228

**Published:** 2026-02-23

**Authors:** Zoé Fremont-Debaene, Suzanne Faure-Dupuy

**Affiliations:** 1 Institut Cochin, INSERM, CNRS, Université Paris Cité, F-75014 Paris, France

**Keywords:** host–pathogen interactions, immune evasion, antiviral treatment, macrophages, viruses

## Abstract

Macrophages are versatile innate immune cells that play a crucial role in immune responses and tissue repair. However, their plasticity and central role in immunity also make them prime targets for viral manipulation. Viruses have evolved sophisticated mechanisms to modulate macrophage functions, disrupting cytokine secretion and phagocytosis to evade immune clearance, establish infection and promote persistence. For instance, some viruses drive excessive cytokine secretion, resulting in hyperinflammation and tissue damage, while other viruses suppress cytokine production and impair phagocytic activity to evade detection. These disruptions often result in systemic immunopathology, chronic inflammation or the establishment of viral reservoirs. Emerging therapeutic strategies aiming to restore macrophage functionality through direct-acting antivirals or macrophage-specific interventions represent a growing interest. These novel approaches offer promising perspectives for combating viral-induced macrophage dysfunction. By summarizing the interplay between viruses and macrophages, this review highlights critical pathways of immune modulation and underscores innovative therapeutic strategies to restore immune balance, offering hope for combating viral infections and their associated pathologies.

## Introduction

Macrophages are indispensable components of the innate immune system, acting as both first responders to infection and orchestrators of long-term immune responses [1]. These cells are involved in a broad range of functions, including pathogen recognition [2], phagocytosis [3], antigen presentation [4] and tissue repair [1]. Their remarkable plasticity enables them to adapt to environmental cues and polarize into distinct functional states [5], ranging from pro-inflammatory functions to anti-inflammatory and tissue-repair-promoting functions. This adaptability is crucial for maintaining tissue homeostasis and combating pathogens. However, this efficacy makes macrophages a prime target for viruses. Indeed, viruses have evolved sophisticated strategies to modulate macrophage functions, enabling them to evade immune surveillance, establish infection and facilitate their own replication and spread. Depending on the virus and the context of infection, macrophages may be paralysed or overactivated [6,7]. For example, some viruses directly interfere with macrophage signalling pathways, such as those governing cytokine production and IFN responses, impairing the host’s ability to mount effective antiviral defences [8]. The consequences of viral modulation of macrophages extend beyond the immediate site of infection. Dysregulated macrophage activity can contribute to systemic immunopathology, chronic inflammation or the establishment of a viral reservoir. For instance, hyperactivation of macrophages is a hallmark of cytokine storm syndromes observed in severe cases of infections such as severe acute respiratory syndrome coronavirus 2 (SARS-CoV-2) [7,9]. Conversely, inhibitions of macrophages’ functions by viruses like human rhinovirus 16 (HRV16) can lead to increased susceptibility to opportunistic infections and disease exacerbations [10,12]. This review aims to provide an overview of some mechanisms by which different viruses modulate macrophage cytokine secretion as well as phagocytosis. By understanding these complex interactions, we can uncover new opportunities for therapeutic interventions, such as restoring normal macrophage function to enhance antiviral immunity.

## Macrophages: the multitaskers of immunity and healing

Macrophages are innate immune cells serving as the first line of defence against pathogens. Their diverse functions include pathogen recognition, cytokine secretion and phagocytosis, which collectively ensure a robust immune response and tissue homeostasis.

### Functions of macrophages

#### Recognition of viruses via PRRs

Macrophages detect pathogens through pattern recognition receptors (PRRs) [2], which recognize conserved microbial structures known as pathogen-associated molecular patterns (PAMPs) or endogenous damage-associated molecular patterns (DAMPs). These PRRs include various types, such as toll-like receptors (TLRs) [13], NOD-like receptors (NLRs) [14] and RIG-I-like receptors (RLRs) [15]. Upon recognition of PAMPs or DAMPs, PRRs initiate complex intracellular signalling cascades that orchestrate immune responses, including antiviral responses, including the production of pro-inflammatory cytokines, type I IFNs and the activation of downstream effector mechanisms like phagocytosis [16] as well as antigen presentation.

For instance, some TLRs such as TLR7 can activate the NF*κ*B signalling pathway [17,18] upon recognition of ssRNA in endosomes, a PAMP associated with viruses. This activation drives, among others, the expression of pro-inflammatory cytokines (such as TNF-*α*, IL-6 and IL-1*β*) [19,20]. Meanwhile, TLR3 recognizes dsRNA in endosomes, a common feature of viral genomes internalized by macrophages [21,22]. TLR3 can activate the IRF3 signalling pathway, leading to the production of type I IFNs that induce the expression of IFN-stimulated genes (ISGs), enhancing the antiviral state of neighbouring cells [23].

Another example of PRRs that play a key role in antiviral responses is RLRs [15] that play an essential role in detecting cytosolic viral RNAs. Retinoic acid-inducible gene I (RIG-I) and melanoma differentiation-associated protein 5 (MDA5) both recognize dsRNAs. RIG-I recognizes short dsRNA with 5′-triphosphate or 5′-diphosphate ends, as well as uncapped RNAs, which are features commonly associated with viral genomes but not host mRNAs [24]. On the other hand, MDA5 primarily detects long dsRNA, a molecular signature commonly associated with viral replication [25]. Upon binding with viral RNA, RIG-I and MDA5 drive the phosphorylation of IRF3 and IRF7 [26,27], leading to the transcription of type I IFNs [28]. RIG-I also triggers IRF9, which participates downstream in IFN alpha/beta receptor signalling as part of the ISGF3 complex, amplifying ISG expression and reinforcing the antiviral state [29]. In parallel, RIG-I activation promotes NF*κ*B signalling, contributing to pro-inflammatory cytokine production.

In addition to RNA-sensing PRRs, macrophages also rely on cytosolic DNA sensors to detect DNA viruses. For instance, the cyclic GMP-AMP synthase (cGAS) recognizes dsDNA that appears in the cytosol during viral infection [30]. Upon DNA binding, the cGAS–STING pathway is activated, resulting in the phosphorylation and activation of IRF3 and activation of the NF*κ*B pathway, resulting in the secretion of type I IFNs and other cytokine genes, establishing a potent defence against DNA viruses [30,31]. Moreover, the inflammasome IFN-*γ*-inducible protein 16 (IFI16) is also able to detect nuclear viral DNA [32]. Upon viral DNA sensing, IFI16 translocates to the cytoplasm, where it can oligomerize and signal through stimulator of IFN genes (STING), similarly to cGAS. IFI16 activation can induce the production of IL-1*β*, as well as STING-dependent IFNs production.

The complementary roles of the different PRRs allow macrophages to detect a broad range of viruses, ensuring a robust antiviral response [33,34].

#### Cytokine secretion

Recognition of PAMPs by TLRs, NLRs or RLRs leads to cytokine secretion that enables macrophages to coordinate immune responses. Upon activation, macrophages produce pro-inflammatory cytokines such as TNF-*α*, IL-6 and IL-1*β* to amplify inflammation, attract immune cells and enhance antimicrobial defences [35]. The transcriptional regulation of these cytokines is controlled by key transcription factors, including NF*κ*B [19] and IFN regulatory factors (IRFs) [36]. Upon stimulation, NF*κ*B transcription factors translocate to the nucleus, where they drive the expression of TNF-*α*, IL-6 and IL-1*β*, promoting immune activation and inflammation. On the other hand, in parallel, IRFs, particularly IRF3 and IRF7, play a crucial role in type I IFNs (IFN-*α*/*β*) production, which is essential for antiviral responses. Conversely, macrophages also produce anti-inflammatory cytokines such as IL-10 and tumour growth factor-beta (TGF-*β*), which resolve inflammation and restore tissue homeostasis [37]. The expression of these cytokines is regulated by transcription factors such as signal transducer and activator of transcription 3 (STAT3) [38] and Krüppel-like factor 4 [39], which promote an anti-inflammatory macrophage phenotype, counterbalancing excessive immune activation.

The inflammasome also plays a key role in cytokine secretion. The best-characterized inflammasome, NLRP3, is activated by a variety of stimuli (ATP, microbial toxins, etc.) [40,41]. After assembling, it recruits and activates caspase-1 that cleaves pro-IL-1*β* and pro-IL-18 into their active forms, leading to the secretion of these potent inflammatory cytokines [42].

#### Phagocytosis

Phagocytosis is a critical process that allows macrophages to capture, internalize and degrade pathogens, apoptotic cells and cellular debris [3]. This multistep process begins with the recognition of targets via PRRs [2] or opsonin receptors, such as Fc*γ* receptors [43] and complement receptors [44]. Upon ligand binding, signalling pathways are activated, leading to localized actin remodelling. This remodelling is mainly driven by the Arp2/3 complex, which nucleates branched actin filaments, forming an actin-rich structure known as the phagocytic cup [45]. The actin cup surrounds the target, facilitating its internalization into a membrane-bound compartment called the phagosome.

After its initial formation, the phagosome will go through a maturation process that involves a series of fusion events with early and late endosomes to end with a fusion with lysosomes to form what is called the phagolysosome [3]. This compartment serves as a site of degradation, utilizing both oxygen-dependent and oxygen-independent mechanisms. Oxygen-dependent mechanisms include the generation of reactive oxygen species (ROS) through the action of NADPH oxidase, which assembles at the phagosomal membrane [46]. Oxygen-independent mechanisms rely on lysosomal enzymes, such as proteases, nucleases and lipases, which degrade the captured material.

In addition to pathogen clearance, macrophages process and present pathogen-derived antigens on major histocompatibility complex class II (MHC-II) molecules to activate T cells and bridge innate and adaptive immunity [47]. This dual role of phagocytosis in degradation and antigen presentation underscores its importance in maintaining immune homeostasis and coordinating an effective immune response.

### The heterogeneity of macrophages

Macrophages exhibit remarkable heterogeneity, influenced by their origin and tissue-specific environments.

#### Origins and tissue-specificity of macrophages

Tissue-resident macrophages arise from two main sources: foetal-derived macrophages and monocyte-derived macrophages (MDMs). Foetal-derived macrophages originate during embryogenesis from yolk sac or foetal liver progenitors and persist into adulthood through self-renewal, maintaining homeostasis in several tissues, including the brain (microglia), liver (Kupffer cells) and lungs (alveolar macrophages) [1]. In contrast, MDMs differentiate from blood-circulating monocytes that infiltrate tissues in response to inflammatory signals or during steady-state conditions, contributing to macrophage heterogeneity. Some tissues, such as the gut, rely heavily on MDM replenishment, while others, like the brain, primarily sustain their macrophage populations through self-renewal [1].

However, in organs where macrophages are predominantly foetal-derived, severe depletion due to infection or inflammation can lead to the replacement of resident macrophages by MDMs, which can acquire a tissue-resident phenotype [1]. This process has been observed in the liver, where monocyte-derived cells can differentiate into Kupffer-like macrophages [48].

Macrophages adapt to their local microenvironment, acquiring specialized roles in response to tissue-specific cues that drive their differentiation and function. This heterogeneity is shaped by a combination of cytokine signalling, cellular interactions and metabolic factors that influence macrophage identity in different organs [1].

The diverse signalling pathways governing the development of resident macrophage populations emphasize their tissue-specific phenotypes and underscore the resulting functional heterogeneity.

#### Phenotypes and plasticity of macrophages

Macrophages exhibit remarkable plasticity, enabling them to continuously adapt their functional states in response to environmental stimuli [49]. This dynamic range of phenotypes exists along a continuum, rather than distinct pro-inflammatory or anti-inflammatory states, allowing macrophages to finely tune immune responses depending on the physiological context. Their adaptability is essential for maintaining immune balance, responding to infections, resolving inflammation and promoting tissue repair [50].

During infections, macrophages adopt an inflammatory phenotype characterized by the production of pro-inflammatory cytokines, as well as the release of ROS and nitric oxide (NO) to eliminate pathogens [51,52]. These macrophages upregulate markers such as CD80, CD86 and MHC-II, which enhance antigen presentation and T cell activation [53,54].

As the infection is cleared, macrophages gradually shift towards a resolution and repair phenotype [55], which supports tissue regeneration and the restoration of homeostasis [56]. This transition is marked by the secretion of anti-inflammatory cytokines, as well as growth factors, which contribute to wound healing, extracellular matrix remodelling and angiogenesis [57]. These macrophages often express markers associated with phagocytosis of apoptotic cells (efferocytosis) and suppression of excessive inflammation [58].

This functional plasticity highlights the critical role of macrophages in orchestrating immune responses while maintaining tissue integrity [50]. Their ability to integrate diverse signals from their microenvironment ensures a balanced immune response, adapting dynamically to changing physiological and pathological conditions.

## Macrophages under siege: viral strategies to subvert immunity

As the first line of defence, macrophages are often the target of viral infections. Here, we present a non-exhaustive list of viruses that can modulate macrophage functions, more specifically, cytokine secretion and phagocytosis.

### Induction of cytokine secretion

Viruses can exploit macrophages to facilitate their establishment and/or replication in the host, often triggering excessive cytokine production and secretion. While pro-inflammatory cytokines play critical roles in immune and antiviral responses, uncontrolled or sustained production contributes to tissue damage and systemic inflammation [9,59].

#### Activation of the NF*κ*B pathway and cytokine secretion

As mentioned before, several viral families can be recognized by PRRs, triggering cytokine production and secretion in macrophages through NF*κ*B activation, a crucial signalling hub for producing pro-inflammatory cytokines, as it integrates multiple upstream signals from PRRs, including TLRs and RLR. Its involvement across multiple viral infections highlights its importance in innate immunity and makes it an attractive therapeutic target.

Viral RNAs are often the trigger of NF*κ*B signalling and inflammatory responses in macrophages. Those from Orthoflaviviruses, such as dengue virus (DENV) [60], Zika virus (ZIKV) [61] and West Nile virus (WNV) [62], as well as the respiratory viruses influenza A virus (IAV) [63,65] and SARS-CoV-2 [66], can activate TLR3 or TLR7 upon viral entry and replication, leading to NF*κ*B activation and subsequent secretion of pro-inflammatory cytokine.

Moreover, it was shown that, indirectly, the inflammatory mediators produced during epithelial cell infection by SARS-CoV-2 can stimulate primary human macrophages to enhance cytokine production and to drive cellular activation [67]. However, recent work also showed that antibodies against the SARS-CoV-2 spike receptor-binding domain can mediate productive infection of primary human macrophages via Fc receptors. Macrophage infection then leads to viral recognition and high levels of IL-6, CXCL9, CXCL10 and IFN-*α* production [68], suggesting a potent activation of NF*κ*B signalling, among others. This emphasizes that macrophages can be modulated by either direct or indirect mechanisms during SARS-CoV-2 infection. Therefore, whereas cytokine release in macrophages can be linked to severe COVID-19 forms, macrophage-derived cytokines can in turn restrain viral spread in bystander cells, releasing viral burden.

**Fig. 1.**
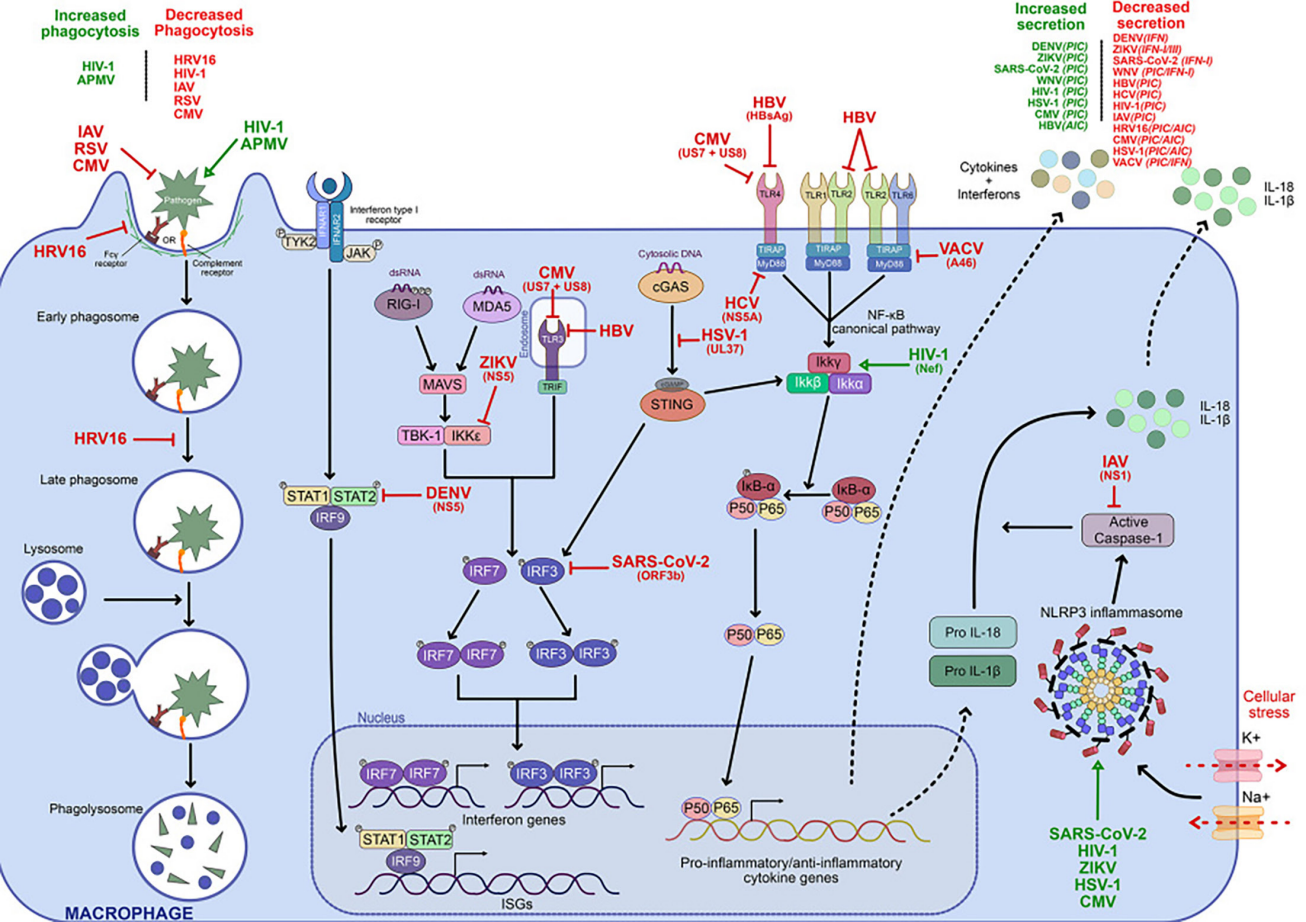
Immunological functions of macrophages modulated by viral pathogens. The recognition of pathogens via different cellular receptors such as PRRs (NRLs, TLRs), Fc*γ* receptors and complement receptors can trigger phagocytosis (*left side of the scheme*), which is the uptake, internalization and degradation of pathogens. Another function triggered by the recognition of pathogens is the production of pro/anti-inflammatory cytokines and IFNs via intracellular signalling cascades such as the NF*κ*B and IRF pathways (*middle of the scheme*). Type I IFNs engage their surface receptors and activate the JAK-STAT signalling pathway (*middle of the scheme*). Then, STAT proteins are phosphorylated, dimerize and translocate to the nucleus, where they induce the expression of ISGs, reinforcing the antiviral state of the cell. In parallel, activation of the NLRP3 inflammasome (*right side of the scheme*) after cellular stress results in caspase-1 activation and the secretion of IL-1*β* and IL-18, amplifying the inflammatory response. Several viruses (and the viral protein involved when it is known) are positioned along specific signalling pathways, where they act as activators or inhibitors of macrophage functions, illustrating the diverse viral strategies to evade or manipulate the host immune response. Legend for arrows: Full black arrows: activation/continuation of signalling/phagocytic pathways. Dashed black arrows: translation or secretion of cytokines/IFNs. Green arrows: viral activation of a pathway or function. Red arrows: viral inhibition of a pathway or function. Acronyms: TYK (tyrosine-protein kinase), cGAMP (cyclic GMP-AMP), MAVS (mitochondrial antiviral signalling protein), TBK-1 (TANK-binding kinase 1), TRIF (TIR-domain-containing adapter-inducing IFN-*β*), TIRAP (TIR domain-containing adaptor protein), Myd88 (Myeloid differentiation primary response 88), K (potassium), Na (sodium), NLRP3 (NOD-like receptor family, pyrin domain containing 3), IFNAR (IFN-*α*/*β* receptor), APMV (Acanthamoeba polyphaga mimivirus), PIC (pro-inflammatory cytokines), AIC (anti-inflammatory cytokines).

Importantly, beyond passive recognition of viral RNA, some viruses encode proteins that actively modulate macrophage cytokine secretion. For example, DENV NS1 protein interacts with high-density lipoproteins (HDL) [69] on macrophages, amplifying pro-inflammatory cytokine release. This dysregulated activation has been associated with progression towards cytokine storm, a hallmark of severe dengue disease [59,70]. Moreover, recent transcriptomic and functional analyses of macrophages infected with DENV show dynamic inflammatory response, with an early wave of NF*κ*B-dependent cytokine gene induction. This is followed by a second, stronger inflammatory phase coinciding with peak viral replication and high secretion of TNF-*α*, IL-6 and chemokines. These data directly link productive macrophage infection by DENV to the dynamics of cytokine storm in severe dengue [71]. Similarly to DENV, human immunodeficiency virus-1 (HIV-1), through the Nef protein, can enhance NF*κ*B signalling activation by promoting the activation of upstream kinases like I*κ*B kinase (IKK) [72,73] (Fig. 1).

DNA viruses can also induce NF*κ*B-dependent cytokine secretion in macrophages, although this is sometimes more transient. For instance, cytomegalovirus (CMV) US31 protein activates NF*κ*B in newly infected monocytes and differentiating macrophages, leading to production of pro-inflammatory cytokines and chemokines [74]. Notably, excessive or sustained secretion of such cytokines has been strongly associated with the development and exacerbation of systemic autoimmune diseases such as systemic lupus erythematosus [75,77].

Finally, even though hepatitis B virus (HBV) does not replicate in macrophages, this DNA virus can indirectly modulate macrophage cytokine profiles. HBV exposure leads to the activation of the NF*κ*B pathway, resulting in the secretion of pro-inflammatory cytokines at early times post-exposure [78]. However, in the long run, it also increases the production of anti-inflammatory cytokines such as IL-10 and TGF-*β* by liver macrophages [79], thereby skewing the response towards an immunoregulatory phenotype (Fig. 1).

Together, these observations highlight that NF*κ*B activation in macrophages is a common target across viral families and can sometimes lead to pathologies linked with hyperinflammation.

#### Inflammasome activation

In addition to NF*κ*B activation, many viruses also trigger inflammasome activation, leading to the release of the potent pro-inflammatory cytokines IL-1*β* and IL-18. A first look at RNA viruses shows that SARS-CoV-2 activates the NLRP3 inflammasome in macrophages, contributing to systemic inflammation [80,82] (Fig. 1). Indeed, several viral proteins, including ORF3a and the envelope (E) protein, have been implicated in this process, and infected macrophages can undergo pyroptosis, releasing DAMPs that further exacerbate the inflammation. However, while these proteins clearly activate inflammasomes in experimental systems, their precise contribution to the cytokine storm that is central to the multi-organ failure and systemic inflammation characteristic of severe COVID-19 is still unknown [83].

HIV-1 also induces inflammasome activation [84] (Fig. 1): MDMs exposed to HIV-1 activate NLRP3 and NAIP/NLRC4 pathways, leading to IL-1*β* and IL-18 release. Similarly, ZIKV [85,87] infection facilitates NLRP3 inflammasome assembly and IL-1*β* secretion in macrophages (Fig. 1). However, some studies also indicate that the viral NS3 protein can impair NLRP3 activation in specific contexts [85], emphasizing that viruses can both activate and dampen inflammasomes depending on the stage of infection and the cellular environment.

DNA viruses can also modulate inflammasomes in macrophages. Herpes simplex virus-1 (HSV-1) triggers NLRP3 inflammasome activation [88] (Fig. 1). CMV has likewise been reported to induce NLRP3 activation in monocytes/macrophages, contributing to systemic inflammation in vulnerable hosts [89] (Fig. 1).

The modulation of macrophage cytokine production by viruses is not just a reflection of the immune response but also has significant pathological implications. For example, the severe symptoms of orthoflavivirus infection, such as haemorrhagic fevers, are primarily due to the overactivation of the inflammatory response that can lead to a cytokine storm [59,90,92].

In summary, viruses may activate macrophages by different mechanisms, including, but not limited to, PRRs and subsequent NF*κ*B signalling and/or inflammasome signalling. For the above-mentioned viruses, this activation leads to the aberrant secretion of pro-inflammatory cytokines. Whereas these immune responses are critical for viral defence, their excess critically contributes to the development of pathogenesis. The delicate balance that exists between immune activation and immune-mediated pathogenesis would need to be carefully restored to avoid the deterioration of the tissue while allowing an efficient antiviral immune response.

### Inhibition of cytokine secretion

Whereas some viruses induce abnormal macrophage cytokine production leading to immune pathogenesis, others can suppress it to evade immune detection and limit antiviral effector functions.

#### Inhibition of PRR activation and NF*κ*B signalling pathways

As mentioned before, TLR activation and NF*κ*B signalling are key components of antiviral immune responses. To evade this immune response, many viruses have evolved strategies to inhibit NF*κ*B activation and cytokine secretion by macrophages.

Several viruses modulate the level of PRRs expression or localization. CMV proteins US7 and US8 bind to both TLR3 and TLR4 promoting their degradation via proteasomal or lysosomal pathways and thereby limiting macrophage responsiveness to viral and bacterial stimuli [93] (Fig. 1). HBV similarly downregulates the expression of key PRRs in liver macrophages, particularly TLR2 and TLR3, which are critical for recognizing HBV capsid and genome, respectively [94,95] (Fig. 1). This is particularly of interest considering that HBV does not replicate in these cells, suggesting that the dampening of potent immune responses by macrophages favours viral establishment and/or maintenance. In addition, HBV inhibits the production of pro-inflammatory cytokines such as IL-6 and IL-1*β* following TLR4 or inflammasome stimulation, a mechanism that depends on HBsAg [79,96] (Fig. 1). These CMV- and HBV-mediated effects contribute to immune evasion and persistence of the viral infection in the host [79,97].

Other viruses target downstream signalling components. For example, hepatitis C virus (HCV) inhibits IL-6 production upon TLR4 stimulation in macrophage cell lines via an NS5A-MyD88-dependent mechanism [8]. This interferes with TLR2, TLR4, TLR7 and TLR9 signalling, dampening antiviral immune responses (Fig. 1). Importantly, as HCV replicates in macrophages [98], TLR7 signalling inhibition can prevent direct antiviral responses. Vaccinia virus (VACV) and other poxviruses encode A46 protein that binds TLR adaptors and prevents activation of NF*κ*B and IRFs, thereby broadly attenuating cytokine and IFN responses in macrophages [99,100] (Fig. 1).

Some viruses also directly interfere with NF*κ*B activation. HIV-1 provides an example of dynamic NF*κ*B modulation: while Nef can enhance NF*κ*B activation at early stages of infection, as stated before, more recent studies indicate that Nef and Vpu may also act as suppressors of NF*κ*B-mediated transcription [101]. Although these observations were made in CD4^+^ T cells in which both proteins reduced pro-inflammatory cytokine production during chronic infection, it is plausible that similar mechanisms operate in macrophages, which, like CD4^+^ T cells, serve as important reservoirs for HIV-1 [102,103].

Interestingly, influenza A NS1 protein controls caspase-1, normally activated by the active NLRP3 inflammasome, thus blocking the cleavage of pro-IL-1*β* and pro-IL-18 and, subsequently, their secretion [104] (Fig. 1).

Finally, some viruses also employ unique but less well-defined mechanisms. For example, whereas HRV16 does not replicate in macrophages [105], it inhibits cytokine production in response to secondary bacterial infections, though this mechanism is still under investigation [6].

#### Inhibition of type I IFN responses

Type I IFNs are crucial for controlling viral infections by inducing ISGs with direct or indirect antiviral effects. Unsurprisingly, many viruses have evolved mechanisms to block type I IFN responses.

Several RNA viruses act at the level of IRF activation or Janus kinase/signal transducer and activator of transcription (JAK-STAT) signalling. While ZIKV enhances cytokine secretion, it also suppresses type I IFN production via its NS5 protein, which interacts with IKK*ε* and prevents IRF3 phosphorylation [106] (Fig. 1). Although these findings were not directly obtained in macrophages, given the central role of macrophages in ZIKV dissemination [107], it is plausible that similar mechanisms operate in these cells, potentially contributing to impaired activation of type I IFNs. Similarly, SARS-CoV-2 ORF3b inhibits IRF3 activation, suppressing type I IFN production [108,109] (Fig. 1), but evidence specifically in macrophages remains limited. Another example is WNV that interferes with JAK-STAT signalling in primary human macrophages, leading to reduced STAT1 activation and attenuated production of type I IFN production [110]. Additionally, it was shown that DENV NS5 binds STAT2, preventing its phosphorylation and thereby blocking IFN-*α* signalling and ISG induction [111] (Fig. 1). However, these results were not directly tested on macrophages, but once again, since macrophages play a key role in DENV infection, it is not excluded that this effect could be extended to macrophages.

DNA viruses can also inhibit IFN response. For instance, HSV-1 encodes a protein (UL37) that deaminates cGAS, which impairs its ability to catalyse cyclic GMP-AMP synthesis and blocks the activation of the STING signalling pathway. This prevents IRF3 activation and IFN-*β* production in infected myeloid cells [112] (Fig. 1). HBV also contributes indirectly to type I IFN suppression in macrophages by inducing IL-10 and TGF-*β* [79], which are potent inhibitors of IFN-*β* production [113].

While significant progress has been made in understanding the interactions between macrophages and viruses when it comes to cytokine secretion, as shown with this non-exhaustive list, many mechanisms remain elusive. Given the central role of NF*κ*B, inflammasome signalling and type I IFN responses in antiviral responses, future research should explore therapeutic strategies that fine-tune these pathways to mitigate viral pathogenesis without compromising antiviral immunity.

However, beyond the molecular mechanisms described above, it is also important to distinguish whether macrophages act as direct viral targets or as bystander cells. In some infections, such as DENV [114] or HIV-1 [102], macrophages can be productively infected and contribute directly to viral replication and cytokine release, whereas in others, such as HBV, macrophages may respond primarily to viral products and infected epithelial cells without being themselves significantly infected. Selected examples of these different scenarios and their impact on disease outcome are summarized in Table 1.

**Table 1.** Examples of macrophages acting as direct viral targets versus bystander responders Representative viruses that either productively infect macrophages or activate them as bystander cells through sensing of viral PAMPs or inflammatory signals. For each virus, we indicate the macrophage infection status, the main pathways modulated (e.g. NF*κ*B, inflammasomes, cGAS–STING, phagocytosis) and the consequences for disease severity or persistence.

Virus	Family	Genome	Macrophages	Increased cytokine secretion	Decreased cytokine/IFN secretion	Effect on phagocytosis	Potential pathological consequences
CMV	*Herpesviridae*	DNA	Monocytes infected	NF*κ*B activation, NLRP3 activation	TLR signalling inhibition	Decreased phagocytosis	Immune evasion, chronic inflammation
DENV	*Flaviviridae*	RNA	Productively infected	NF*κ*B activation, interaction with HDL	JAK-STAT pathway inhibition	–	Cytokine storm, haemorrhagic fever
HBV	*Hepadnaviridae*	DNA	Bystander	NF*κ*B activation (early-stage post-exposure)	TLR signalling inhibition, IL-10 and TGF-*β* induced inhibition of IFN-I production	–	Persistence
HCV	*Flaviviridae*	RNA	Bystander	–	TLR signalling inhibition	–	Chronic infection
HIV-1	*Retroviridae*	RNA	Productively infected	NF*κ*B activation (early stage of infection), NLRP3 activation	NF*κ*B inhibition	Increased/decreased phagocytosis	Viral reservoir
HRV16	*Picornaviridae*	RNA	Bystander	–	Unknown mechanism	Decreased phagocytosis	Bacterial superinfections, COPD exacerbations
HSV-1	*Herpesviridae*	DNA	Productively infected	NF*κ*B activation, NLRP3 activation	cGAS–STING inhibition	–	Local inflammation
IAV	*Orthomyxoviridae*	RNA	Abortively infected	NF*κ*B activation	NLRP3 inhibition	–	Lung pathology
Mimivirus	*Mimiviridae*	DNA	Productively phagocytosed	–	–	Increased phagocytosis	Viral dissemination, pneumonia
SARS-CoV-2	*Coronaviridae*	RNA	Mostly bystander	NF*κ*B activation, NLRP3 activation	IRF3 pathway inhibition	–	Cytokine storm, severe COVID
RSV	*Pneumoviridae*	RNA	Abortive	Dysregulated IFN-*β*	–	Decreased phagocytosis	Risk of bacterial superinfections
VACV	*Poxviridae*	DNA	Bystander	–	TLR signalling inhibition	–	Immune evasion
WNV	*Flaviviridae*	RNA	Productively infected	NF*κ*B activation	IRF3 pathway inhibition	–	Neuroinflammation
ZIKV	*Flaviviridae*	RNA	Productively infected	NF*κ*B activation, NLRP3 activation	IRF3 pathway inhibition	–	Tissue inflammation, neuropathology

### Modulation of phagocytosis

The modulation of macrophage phagocytosis by viruses is a key strategy for immune evasion and viral persistence. By impairing the phagocytic activity of macrophages, viruses can prevent the effective clearance of viral particles or infected cells and modulate the host immune response to favour their replication. Different viruses utilize distinct mechanisms to interfere with phagocytosis, such as altering receptor signalling, inhibiting actin dynamics or disrupting autophagic pathways. These viral strategies not only enhance viral survival but also contribute to the pathogenesis of various diseases, ranging from chronic infections to increased susceptibility to secondary bacterial infections.

When it comes to altering phagocytosis, viruses can be divided into two: those who modulate phagocytosis without an evident benefit for the virus and those who do it to enable viral entry or increase viral replication.

The first category of viruses has been shown to either block key immune receptors, manipulate the macrophage’s cytoskeletal dynamics or impair macrophage differentiation to reduce the efficiency of phagocytosis. Infection of macrophages by IAV or respiratory syncytial virus (RSV) decreases their phagocytic capacity [115] (Fig. 1). This phenomenon has been attributed to an autologous release of IFN-*β* [115]. HRV16 can also impair phagocytosis by impairing actin remodelling in a mechanism dependent on the Arpin protein, a negative regulator of the Arp2/3 complex [116] (Fig. 1). This blocks the formation of the phagocytic cup and impairs bacterial uptake and receptor‐mediated phagocytosis in macrophages [116]. Additionally, HRV16 impairs phagosome maturation in an ARL5b-dependent mechanism, allowing bacteria survival in the macrophages [117]. Interestingly, induction of ARL5b seems to be a side effect of HRV16 exposure, as it is dependent on the viral receptor ICAM1 and subsequent activation of PKR and ATF2 [118] (Fig. 1). By interfering with macrophage phagocytosis, HRV16 can lead to bacterial superinfections that are often associated with severe COPD exacerbations [119,120]. Interestingly, CMV infection of macrophages alters their differentiation, reducing their phagocytic index and impairing bacterial clearance [121,122] (Fig. 1).

The second category of viruses manipulates macrophage phagocytosis to enhance their entry into macrophages. For instance, HIV infection alters macrophage interactions with CD4^+^ T cells. The viral accessory protein Vpu has been reported to downregulate CD47 on infected CD4^+^ T cells. CD47 normally delivers a ‘don’t eat me’ signal that prevents phagocytosis by macrophages [123]. Its downregulation enhances the capture and phagocytosis of infected T cells by macrophages [124] (Fig. 1). While this mechanism can be interpreted as a viral strategy to promote infection of macrophages and establishment of long-lived reservoirs, it may also reflect a host-driven antiviral clearance pathway: phagocytosis of infected T cells can lead to degradation of viral material and restriction of viral spread. Yet, in certain contexts, it may still allow the transfer of infectious particles. On the other hand, HIV can also impair macrophage phagocytic function through other mechanisms. HIV-1 infection alters phagosome migration and velocity in primary human macrophages, indicating compromised intracellular trafficking during phagocytosis [125,126] (Fig. 1). Additionally, since HIV-1-infected macrophages exhibit defective phagocytic properties, this may contribute to the development of opportunistic infections such as invasive *Salmonella typhimurium* [127]. Some DNA viruses may also exploit phagocytosis in order to infect the cells more efficiently. It is, for example, the case of Acanthamoeba polyphaga mimivirus [128] (Fig. 1).

In summary, whether viruses target the phagocytosis capacity of macrophages to their advantage or not, the consequences of these modulations can have important adverse effects. Understanding the mechanisms by which viruses modulate macrophage phagocytosis provides valuable insights into viral immune evasion tactics and highlights potential therapeutic targets for improving immune response and controlling viral infections. Re-enabling efficient phagocytosis by macrophages should be considered as a treatment option in different pathologies, including, but not limited to, COPD (Chronic Obstructive Pulmonary Disease) patients.

## Macrophage showdown: therapies to outwit viral hijacking

Since viral exploitation of macrophages can contribute to disease progression, restoring macrophage functionality should be a key focus of therapeutic research. Two main approaches are being explored: therapies that target the virus directly and those that modulate the host’s macrophages. Both strategies hold promises for mitigating immune dysregulation and improving disease outcomes. A non-exhaustive overview of antiviral and host-directed therapies that modulate macrophage functions, together with their clinical status, is provided in Table 2.

**Table 2.** Current and prospective therapies to restore macrophage function in the context of viral infections Major classes of antiviral strategies are summarized with their mechanisms of action, example molecules, their effects on macrophage antiviral functions and their clinical development status. These approaches either reduce viral-induced immune dysregulation indirectly or specifically restore macrophage antiviral activity. Corresponding references in the text are indicated in bold.

Therapy	Mechanism	Examples	Effect on macrophages	Clinical status
DAAs blocking the viral cycle	Inhibit viral replication or entry	HCV: Sofosbuvir (**129**), Velpatasvir (**129**), Voxilaprevir (**129**); SARS-CoV-2: Remdesivir (**120**); CMV: Ganciclovir (**132**); SARS-CoV-2: Casirivimab/Imdevimab (**131**)	Indirect restoration of function, reduced immune suppression/inflammation	Approved for HCV, COVID-19, CMV depending on molecule
DAAs targeting macrophage-specific pathways	Block virus–macrophage interactions or enhance immune signalling	Influenza: Oseltamivir (**134**); HBV, HCV: IFN-*α* (**135, 136**); HIV: Maraviroc (**137**); CSF1R inhibitors (**138**)	Prevent viral evasion of macrophages, maintain or restore immune activity	Oseltamivir, IFN-*α* and Maraviroc are approved; IFN-based combinations standard of care in earlier HBV/HCV regimens
Host-directed immunomodulators	Reprogramme or enhance macrophage activity	SARS-CoV-2: GM-CSF agonists/antagonists (**142, 143**); TLR agonists (**144**) (e.g. TLR2–HBV)	Boost antiviral response; modulate inflammation; reduce viral reservoir	GM-CSF-based treatments in clinical trials; CSF1R inhibition in preclinical/early clinical; TLR agonists mostly preclinical/early clinical in HBV
Epigenetic modulators	Reverse virus-induced epigenetic modulation of immune genes	HDAC inhibitors (**145–147**)	Restore macrophage antiviral gene expression	Several HDAC inhibitors approved in oncology; applications in infections mainly preclinical
Gene-editing therapies	Eliminate latent viral genomes from macrophages	HIV: CRISPR-based strategies (**148**)	Direct removal of viral reservoirs, potential for functional cure	Currently preclinical/proof-of-concept

### Direct-acting antivirals and their impact on macrophages

Direct-acting antivirals (DAAs) remain a cornerstone of antiviral treatment. Their effects on macrophages are mostly indirect: by reducing viral load and limiting immune activation, DAAs help to alleviate virus-induced macrophage dysfunction and restore immune balance. For example, in HCV infection, a combination of NS5B polymerase inhibitors like sofosbuvir, NS5A inhibitors like velpatasvir and NS3/4A protease inhibitors like voxilaprevir effectively block viral replication [129]. This not only suppresses hepatocyte infection but also reduces systemic inflammation, indirectly restoring macrophage responsiveness. Similarly, in SARS-CoV-2 infection, DAAs such as remdesivir [130] inhibit viral RNA synthesis, while neutralizing antibodies like casirivimab and imdevimab prevent viral entry [131], thereby indirectly protecting macrophages from viral-induced immune dysregulation. Another example is ganciclovir, a guanosine analogue that inhibits viral DNA polymerase and is used to treat CMV infection [132]. By reducing viral replication, ganciclovir reverses the immunosuppressive effects of CMV on macrophages.

Beyond general viral replication inhibitors, some DAAs target specific mechanisms that viruses use to impair macrophage function. For instance, influenza viruses exploit receptors expressed by macrophages, such as sialic acid [133], to evade immune responses. Neuraminidase inhibitors like oseltamivir [134] prevent this interaction, allowing macrophages to maintain their immune activity. Additionally, IFN-stimulating agents, such as pegylated IFN-*α*, can enhance macrophage function by countering viral suppression of immune signalling pathways, as observed in HBV [135] and HCV [136] infections. In the context of HIV, CCR5 antagonists such as maraviroc [137] prevent the virus from infecting macrophages by blocking the receptor used for viral entry, while CSF1R inhibitors [138] can reduce macrophage populations harbouring latent viruses. However, while DAAs can be highly effective, they have limitations. Their efficacy is often virus-specific, and the selective pressure they impose on viruses can drive resistance. Additionally, in some cases, controlling viral replication alone is insufficient to fully restore immune homeostasis. This highlights the need for other kinds of therapies that target the host’s macrophages directly.

### Host-directed therapies: a promising alternative

Targeting macrophages directly presents several advantages. Firstly, unlike DAAs, these therapies impose less selective pressure on viruses, reducing the risk of resistance [139,140]. Furthermore, macrophage-targeted strategies can offer broader protection against multiple viruses by modulating the host’s immune system rather than focusing on a single pathogen. One key approach involves reprogramming macrophage activity to enhance their antiviral functions. GM-CSF (Granuloyte Macrophage Colony Stimulating Factor), for example, enhances macrophage activation and inflammation, boosting their ability to combat severe infections. However, GM-CSF also plays a role in hyperinflammatory conditions [141], leading to a dual approach, as during the COVID-19 pandemic, where both GM-CSF agonists and inhibitors were explored as potential therapies [142,143]. Another promising strategy involves blocking key macrophage receptors that viruses exploit for entry. CSF1R inhibitors, for instance, can reduce macrophage populations that harbour latent viruses, offering a way to target viral reservoirs in chronic infections [138]. Another strategy employs TLR agonists, notably TLR2 agonists, which can activate macrophages to produce antiviral cytokines, with applications in HBV therapy, for example [144].

Viruses also frequently induce epigenetic changes to suppress macrophage antiviral responses and establish persistent infections. Therefore, epigenetic modulation has emerged as an area of growing interest for understanding host–pathogen interactions and developing novel therapeutic strategies. For example, histone deacetylase (HDAC) inhibitors can reverse these virus-induced modifications, restoring macrophage functionality and enhancing antiviral immunity [145,146], as is the case in RSV infection [147]. Expanding research in this area could lead to new treatment strategies that counteract viral immune evasion at the epigenetic level. Additionally, cutting-edge genetic tools like CRISPR-based therapies are being explored to directly excise viral genomes from reservoir cells [148,150]. This approach is particularly relevant for persistent infections such as HIV, where latent viral reservoirs in macrophages pose a major barrier to cure. However, while DAAs can modulate macrophage activation, it is important to note that their effect can also be indirect, resulting from a decrease in viral load rather than direct modulation of macrophage functions.

### The broader utility of macrophage-targeted therapies

Beyond their role in direct antiviral defence, macrophage-targeted therapies offer broader applications in infectious disease treatment. One striking example is the challenge posed by antibody-dependent enhancement (ADE) in orthoflavivirus infections [151,152]. ADE occurs when pre-existing antibodies enhance viral entry into macrophages, exacerbating disease severity. This has been observed in DENV infections, where prior exposure or vaccination can lead to more severe secondary infections [153]. Given these complexities, relying solely on vaccines or DAAs may not be sufficient, and therapies that directly modulate macrophage responses could provide a crucial alternative. By fine-tuning macrophage activation and preventing their exploitation by viruses, host-directed therapies could help mitigate ADE-related complications and improve vaccine efficacy.

## Conclusion

The study of macrophage modulation by viruses is a critical area of research with significant potential for advancing antiviral therapies. As key components of the innate immune system, macrophages play a dual role in initiating and regulating immune responses. Their ability to recognize pathogens, secrete cytokines and perform phagocytosis is essential for host defence. However, this same plasticity that enables them to adapt to diverse environmental cues also makes them susceptible to viral hijacking. Viruses have evolved different mechanisms to manipulate macrophage functions, facilitating immune evasion, enhancing replication and contributing to systemic pathologies such as chronic inflammation or cytokine storms. These interactions are not incidental. They are central to the viral-induced pathogenesis and the progression of many viral infections.

Understanding how viruses modulate macrophage activity by inducing excessive cytokine secretion, impairing phagocytosis or suppressing critical signalling pathways provides valuable insights into immune dysregulation in viral diseases. Investigating these mechanisms is essential not only for unravelling disease mechanisms but also for identifying novel therapeutic targets.

Targeting virus–macrophage interactions offers a promising avenue for therapeutic innovation. Beyond individual viral infections, research into macrophage plasticity and its manipulation by viruses has broader implications for immune resilience. By deepening our knowledge in this field, we can develop strategies to mitigate chronic inflammation, restore immune homeostasis and pave the way for more effective treatments against infectious diseases.

## Abbreviations

ADE: antibody-dependent enhancement
cGAS: cyclic GMP-AMP synthase
CMV: cytomegalovirus
COPD: chronic obstructive pulmonary disease
DAAs: direct-acting antivirals
DAMPs: damage-associated molecular patterns
DENV: dengue virus
GM-CSF: granulocyte macrophage colony stimulating factor
HBV: hepatitis B virus
HCV: hepatitis C virus
HDAC: histone deacetylase
HDL: high-density lipoproteins
HIV-1: human immunodeficiency virus-1
HRV16: human rhinovirus 16
HSV-1: herpes simplex virus-1
IAV: influenza A virus
IFI16: IFN-*γ*-inducible protein 16
IFN: interferon
IKK: I*κ*B kinase
IRFs: IFN regulatory factors
ISGs: IFN-stimulated genes
JAK-STAT: Janus kinase/signal transducer and activator of transcription
MDA5: melanoma differentiation-associated protein 5
MDMs: monocyte-derived macrophages
MHC-II: major histocompatibility complex class II
NLRs: NOD-like receptors
PAMPs: pathogen-associated molecular patterns
PRRs: pattern recognition receptors
RIG-I: retinoic acid-inducible gene I
RLRs: RIG-I-like receptors
ROS: reactive oxygen species
RSV: respiratory syncytial virus
SARS-CoV-2: severe acute respiratory syndrome coronavirus 2
STING: stimulator of IFN genes
TGF-*β*: tumour growth factor-beta
TLRs: toll-like receptors
VACV: vaccinia virus
WNV: West Nile virus
ZIKV: Zika virus

## References

[1] Guan F, Wang R, Yi Z, Luo P, Liu W (2025). Tissue macrophages: origin, heterogenity, biological functions, diseases and therapeutic targets. *Sig Transduct Target Ther*.

[2] Li D, Wu M (2021). Pattern recognition receptors in health and diseases. *Sig Transduct Target Ther*.

[3] Depierre M, Jacquelin L, Niedergang F, Bradshaw RA, Hart GW, Stahl PD, Bradshaw RA, Hart GW (2023). Encyclopedia of Cell Biology.

[4] Muntjewerff EM, Meesters LD, van den Bogaart G (2020). Antigen cross-presentation by macrophages. Front Immunol.

[5] Luo M, Zhao F, Cheng H, Su M, Wang Y (2024). Macrophage polarization: an important role in inflammatory diseases. Front Immunol.

[6] Jubrail J, Africano-Gomez K, Herit F, Baturcam E, Mayer G (2018). HRV16 impairs macrophages cytokine response to a secondary bacterial trigger. Front Immunol.

[7] Wendisch D, Dietrich O, Mari T, von Stillfried S, Ibarra IL (2021). SARS-CoV-2 infection triggers profibrotic macrophage responses and lung fibrosis. Cell.

[8] Abe T, Kaname Y, Hamamoto I, Tsuda Y, Wen X (2007). Hepatitis C virus nonstructural protein 5A modulates the toll-like receptor-myd88-dependent signaling pathway in macrophage cell lines. J Virol.

[9] Gürsoy B, Sürmeli CD, Alkan M, Satıcı C, Altunok ES (2021). Cytokine storm in severe COVID-19 pneumonia. J Med Virol.

[10] Sim YS, Lee JH, Lee EG, Choi JY, Lee C-H (2022). COPD exacerbation-related pathogens and previous COPD treatment. JCM.

[11] Zhu J, Mallia P, Footitt J, Qiu Y, Message SD (2020). Bronchial mucosal inflammation and illness severity in response to experimental rhinovirus infection in COPD. J Allergy Clin Immunol.

[12] Seo H, Sim YS, Min KH, Lee JH, Kim B-K (2022). The relationship between comorbidities and microbiologic findings in patients with acute exacerbation of chronic obstructive pulmonary disease. COPD.

[13] Krutzik SR, Tan B, Li H, Ochoa MT, Liu PT (2005). TLR activation triggers the rapid differentiation of monocytes into macrophages and dendritic cells. Nat Med.

[14] Awad F, Assrawi E, Jumeau C, Georgin-Lavialle S, Cobret L (2017). Impact of human monocyte and macrophage polarization on NLR expression and NLRP3 inflammasome activation. PLoS One.

[15] Wang Y, Feng H, Li X, Ruan Y, Guo Y (2024). Dampening of ISGylation of RIG-I by ADAP regulates type I interferon response of macrophages to RNA virus infection. PLoS Pathog.

[16] Duan T, Du Y, Xing C, Wang HY, Wang R-F (2022). Toll-like receptor signaling and its role in cell-mediated immunity. Front Immunol.

[17] Liu AR, Sarkar N, Cress JD, de Jesus TJ, Vadlakonda A (2024). NF-κB c-Rel is a critical regulator of TLR7-induced inflammation in psoriasis. EBioMedicine.

[18] Leibler C, Thomas KB, Josensi C, Levack RC, Smita S (2025). Divergent TIR signaling domains in TLR7 and TLR9 control opposing effects on systemic autoimmunity. J Clin Invest.

[19] Guo Q, Jin Y, Chen X, Ye X, Shen X (2024). NF-κB in biology and targeted therapy: new insights and translational implications. Sig Transduct Target Ther.

[20] Suh HN, Kim YK, Lee JY, Kang G-H, Hwang JH (2021). Dissect the immunity using cytokine profiling and NF-kB target gene analysis in systemic inflammatory minipig model. PLoS One.

[21] Zhu J, Smith K, Hsieh PN, Mburu YK, Chattopadhyay S (2010). High-throughput screening for TLR3-IFN regulatory factor 3 signaling pathway modulators identifies several antipsychotic drugs as TLR inhibitors. J Immunol.

[22] Jiang Z, Mak TW, Sen G, Li X (2004). Toll-like receptor 3-mediated activation of NF-κB and IRF3 diverges at Toll-IL-1 receptor domain-containing adapter inducing IFN-β. Proc Natl Acad Sci USA.

[23] Zhu H, Hou P, Chu F, Li X, Zhang W (2024). PBLD promotes IRF3 mediated the type I interferon (IFN-I) response and apoptosis to inhibit viral replication. Cell Death Dis.

[24] Wang B, Wang Y, Pan T, Zhou L, Ran Y (2025). Targeting a key disulfide linkage to regulate RIG-I condensation and cytosolic RNA-sensing. Nat Cell Biol.

[25] Pichlmair A, Schulz O, Tan C-P, Rehwinkel J, Kato H (2009). Activation of MDA5 requires higher-order RNA structures generated during virus infection. J Virol.

[26] Kuo R-L, Kao L-T, Lin S-J, Wang RY-L, Shih S-R (2013). MDA5 plays a crucial role in enterovirus 71 RNA-mediated IRF3 activation. PLoS One.

[27] Wan Q, Yang C, Rao Y, Liao Z, Su J (2017). MDA5 induces a stronger interferon response than RIG-I to GCRV infection through a mechanism involving the phosphorylation and dimerization of IRF3 and IRF7 in CIK cells. Front Immunol.

[28] Baños-Lara MDR, Ghosh A, Guerrero-Plata A (2013). Critical Role of MDA5 in the interferon response induced by human metapneumovirus infection in dendritic cells and *In Vivo*. J Virol.

[29] Burkart SS, Schweinoch D, Frankish J, Sparn C, Wüst S (2023). High-resolution kinetic characterization of the RIG-I-signaling pathway and the antiviral response. Life Sci Alliance.

[30] Yum S, Li M, Fang Y, Chen ZJ (2021). TBK1 recruitment to STING activates both IRF3 and NF-κB that mediate immune defense against tumors and viral infections. Proc Natl Acad Sci USA.

[31] Gui X, Yang H, Li T, Tan X, Shi P (2019). Autophagy induction via STING trafficking is a primordial function of the cGAS pathway. Nature.

[32] Unterholzner L, Keating SE, Baran M, Horan KA, Jensen SB (2010). IFI16 is an innate immune sensor for intracellular DNA. Nat Immunol.

[33] Onomoto K, Onoguchi K, Yoneyama M (2021). Regulation of RIG-I-like receptor-mediated signaling: interaction between host and viral factors. Cell Mol Immunol.

[34] Sampaio NG, Chauveau L, Hertzog J, Bridgeman A, Fowler G (2021). The RNA sensor MDA5 detects SARS-CoV-2 infection. Sci Rep.

[35] Grassin-Delyle S, Abrial C, Salvator H, Brollo M, Naline E (2020). The role of toll-like receptors in the production of cytokines by human lung macrophages. J Innate Immun.

[36] Chakravarty S, Varghese M, Fan S, Taylor RT, Chakravarti R (2024). IRF3 inhibits inflammatory signaling pathways in macrophages to prevent viral pathogenesis. Sci Adv.

[37] Sanin DE, Prendergast CT, Mountford AP (2015). IL-10 production in macrophages is regulated by a TLR-driven CREB-mediated mechanism that is linked to genes involved in cell metabolism. J Immunol.

[38] Hwang S, Park J, Koo S-Y, Lee S-Y, Jo Y (2025). The ubiquitin ligase Pellino1 targets STAT3 to regulate macrophage-mediated inflammation and tumor development. Nat Commun.

[39] Liao X, Sharma N, Kapadia F, Zhou G, Lu Y (2011). Krüppel-like factor 4 regulates macrophage polarization. J Clin Invest.

[40] Frising UC, Ribo S, Doglio MG, Malissen B, van Loo G (2022). Nlrp3 inflammasome activation in macrophages suffices for inducing autoinflammation in mice. EMBO Rep.

[41] Swanson KV, Deng M, Ting JP-Y (2019). The NLRP3 inflammasome: molecular activation and regulation to therapeutics. Nat Rev Immunol.

[42] Xu C, Zhu R, Dai Q, Xu G, Zhang G (2025). The role and mechanism of the NLRP3-IL-1β/IL-18 signaling axis in the progression of sepsis under an aging phenotype. Life Sciences.

[43] Shi Y, Sun Y, Seki A, Rutz S, Koerber JT (2024). A real-time antibody-dependent cellular phagocytosis assay by live cell imaging. J Immunol Methods.

[44] Mularski A, Wimmer R, Arbaretaz F, Goff GL, Depierre M (2023). Dynamin-2 controls actin remodeling for efficient complement receptor 3-mediated phagocytosis. Biol Cell.

[45] Krendel M, Gauthier NC (2022). Building the phagocytic cup on an actin scaffold. Curr Opin Cell Biol.

[46] Lee H-J, Woo Y, Hahn T-W, Jung YM, Jung Y-J (2020). Formation and maturation of the phagosome: a key mechanism in innate immunity against intracellular bacterial infection. Microorganisms.

[47] Guillaume J, Leufgen A, Hager FT, Pabst O, Cerovic V (2023). MHCII expression on gut macrophages supports T cell homeostasis and is regulated by microbiota and ontogeny. Sci Rep.

[48] Scott CL, Zheng F, De Baetselier P, Martens L, Saeys Y (2016). Bone marrow-derived monocytes give rise to self-renewing and fully differentiated Kupffer cells. Nat Commun.

[49] Zhang F, Miao Y, Liu Q, Li S, He J (2020). Changes of pro-inflammatory and anti-inflammatory macrophages after peripheral nerve injury. RSC Adv.

[50] Locati M, Curtale G, Mantovani AD (2020). Diversity, mechanisms, and significance of macrophage plasticity. Annu Rev Pathol.

[51] Palmieri EM, Gonzalez-Cotto M, Baseler WA, Davies LC, Ghesquière B (2020). Nitric oxide orchestrates metabolic rewiring in M1 macrophages by targeting aconitase 2 and pyruvate dehydrogenase. Nat Commun.

[52] Zhao K, Huang Z, Lu H, Zhou J, Wei T (2010). Induction of inducible nitric oxide synthase increases the production of reactive oxygen species in RAW264.7 macrophages. Biosci Rep.

[53] Parker D (2018). CD80/CD86 signaling contributes to the proinflammatory response of *Staphylococcus aureus* in the airway. Cytokine.

[54] Redka DS, Gütschow M, Grinstein S, Canton J (2018). Differential ability of proinflammatory and anti-inflammatory macrophages to perform macropinocytosis. Mol Biol Cell.

[55] Sapudom J, Karaman S, Mohamed WKE, Garcia-Sabaté A, Quartey BC (2021). 3D in vitro M2 macrophage model to mimic modulation of tissue repair. NPJ Regen Med.

[56] Pereira-Lopes S, Tur J, Calatayud-Subias JA, Lloberas J, Stracker TH (2015). NBS1 is required for macrophage homeostasis and functional activity in mice. Blood.

[57] Bohaud C, Johansen MD, Jorgensen C, Kremer L, Ipseiz N (2021). The role of macrophages during mammalian tissue remodeling and regeneration under infectious and non-infectious conditions. Front Immunol.

[58] Schilperoort M, Ngai D, Sukka SR, Avrampou K, Shi H (2023). The role of efferocytosis-fueled macrophage metabolism in the resolution of inflammation. Immunol Rev.

[59] Srikiatkhachorn A, Mathew A, Rothman AL (2017). Immune-mediated cytokine storm and its role in severe dengue. Semin Immunopathol.

[60] Kayesh MEH, Kohara M, Tsukiyama-Kohara K (2021). Recent insights into the molecular mechanism of toll-like receptor response to dengue virus infection. Front Microbiol.

[61] da Silva MHM, Moises RNC, Alves BEB, Pereira HWB, de Paiva AAP (2019). Innate immune response in patients with acute Zika virus infection. Med Microbiol Immunol.

[62] Daffis S, Samuel MA, Suthar MS, Gale M, Diamond MS (2008). Toll-like receptor 3 has a protective role against West Nile virus infection. J Virol.

[63] Le Goffic R, Balloy V, Lagranderie M, Alexopoulou L, Escriou N (2006). Detrimental contribution of the Toll-like receptor (TLR)3 to influenza A virus-induced acute pneumonia. PLOS Pathog.

[64] Li H, Wang A, Zhang Y, Wei F (2023). Diverse roles of lung macrophages in the immune response to influenza A virus. Front Microbiol.

[65] Zhang J, Liu J, Yuan Y, Huang F, Ma R (2020). Two waves of pro-inflammatory factors are released during the influenza A virus (IAV)-driven pulmonary immunopathogenesis. PLoS Pathog.

[66] Bortolotti D, Gentili V, Rizzo S, Schiuma G, Beltrami S (2021). TLR3 and TLR7 RNA sensor activation during SARS-CoV-2 Infection. Microorganisms.

[67] Thorne LG, Reuschl A-K, Zuliani-Alvarez L, Whelan MVX, Turner J (2021). SARS-CoV-2 sensing by RIG-I and MDA5 links epithelial infection to macrophage inflammation. EMBO J.

[68] Pickering S, Wilson H, Bravo E, Perera MR, Seow J (2024). Antibodies to the RBD of SARS-CoV-2 spike mediate productive infection of primary human macrophages. Nat Commun.

[69] Benfrid S, Park K, Dellarole M, Voss JE, Tamietti C (2022). Dengue virus NS1 protein conveys pro‐inflammatory signals by docking onto high‐density lipoproteins. EMBO Reports.

[70] Bhatt P, Varma M, Sood V, Ambikan A, Jayaram A (2024). Temporal cytokine storm dynamics in dengue infection predicts severity. Virus Res.

[71] Valdés-López JF, Tamayo-Molina YS, Fernandez GJ, Hernández-Sarmiento LJ, Velilla PA (2025). Early events in dengue virus infection inducing cytokine storm: The dynamic interplay of pattern-recognition receptors, inflammasome activation, and biphasic NF-κB and STAT1-dependent inflammatory responses in human mononuclear phagocytes. PLOS Negl Trop Dis.

[72] Olivetta E, Percario Z, Fiorucci G, Mattia G, Schiavoni I (2003). HIV-1 Nef induces the release of inflammatory factors from human monocyte/macrophages: involvement of Nef endocytotic signals and NF-kappa B activation. *J Immunol*.

[73] Herbein G, Gras G, Khan KA, Abbas W (2010). Macrophage signaling in HIV-1 infection. Retrovirology.

[74] Guo G, Ye S, Xie S, Ye L, Lin C (2018). The cytomegalovirus protein US31 induces inflammation through mono-macrophages in systemic lupus erythematosus by promoting NF-κB2 activation. Cell Death Dis.

[75] Barakat L, Imillou B, Echchilali K, Moudatir M, Kabli HE (2025). Macrophage activation syndrome in systemic lupus erythematosus: report of 8 cases. J Rheumatol.

[76] Cheng Y, Liu L, Ye Y, He Y, Hu W (2024). Roles of macrophages in lupus nephritis. Front Pharmacol.

[77] Ahamada MM, Jia Y, Wu X (2021). Macrophage polarization and plasticity in systemic lupus erythematosus. Front Immunol.

[78] Boltjes A, van Montfoort N, Biesta PJ, Op den Brouw ML, Kwekkeboom J (2015). Kupffer cells interact with hepatitis B surface antigen in vivo and in vitro, leading to proinflammatory cytokine production and natural killer cell function. J Infect Dis.

[79] Faure-Dupuy S, Delphin M, Aillot L, Dimier L, Lebossé F (2019). Hepatitis B virus-induced modulation of liver macrophage function promotes hepatocyte infection. J Hepatol.

[80] Cerato JA, da Silva EF, Porto BN (2023). Breaking bad: inflammasome activation by respiratory viruses. Biology (Basel).

[81] Siu K-L, Yuen K-S, Castaño-Rodriguez C, Ye Z-W, Yeung M-L (2019). Severe acute respiratory syndrome coronavirus ORF3a protein activates the NLRP3 inflammasome by promoting TRAF3-dependent ubiquitination of ASC. FASEB J.

[82] Rodrigues TS, Zamboni DS (2023). Inflammasome activation by SARS-CoV-2 and its participation in COVID-19 exacerbation. Curr Opin Immunol.

[83] Que Y, Hu C, Wan K, Hu P, Wang R (2022). Cytokine release syndrome in COVID-19: a major mechanism of morbidity and mortality. Int Rev Immunol.

[84] Triantafilou K, Ward CJK, Czubala M, Ferris RG, Koppe E (2021). Differential recognition of HIV-stimulated IL-1β and IL-18 secretion through NLR and NAIP signalling in monocyte-derived macrophages. PLoS Pathog.

[85] Gim E, Shim D-W, Hwang I, Shin OS, Yu J-W (2019). Zika virus impairs Host NLRP3-mediated inflammasome activation in an NS3-dependent Manner. Immune Netw.

[86] He Z, Chen J, Zhu X, An S, Dong X (2018). NLRP3 inflammasome activation mediates zika virus–associated inflammation. J Infect Dis.

[87] Wang W, Li G, De Wu W, Luo Z, Pan P (2018). Zika virus infection induces host inflammatory responses by facilitating NLRP3 inflammasome assembly and interleukin-1β secretion. Nat Commun.

[88] Karaba AH, Figueroa A, Massaccesi G, Botto S, DeFilippis VR (2020). Herpes simplex virus type 1 inflammasome activation in proinflammatory human macrophages is dependent on NLRP3, ASC, and caspase-1. PLoS One.

[89] Xu X, Cai J, Wang X, Lu Y, Guo B (2023). Human cytomegalovirus infection activates NLRP3 inflammasome by releasing mtDNA into the cytosol in human THP-1 cells. Microbiol Immunol.

[90] Pan Y, Cai W, Cheng A, Wang M, Yin Z (2022). Flaviviruses: innate immunity, inflammasome activation, inflammatory cell death, and cytokines. Front Immunol.

[91] Pandey N, Jain A, Garg RK, Kumar R, Agrawal OP (2015). Serum levels of IL-8, IFNγ, IL-10, and TGF β and their gene expression levels in severe and non-severe cases of dengue virus infection. Arch Virol.

[92] Chaudhary R, Meher A, Krishnamoorthy P, Kumar H (2023). Interplay of host and viral factors in inflammatory pathway mediated cytokine storm during RNA virus infection. Curr Res Immunol.

[93] Park A, Ra EA, Lee TA, Choi H jin, Lee E (2019). HCMV-encoded US7 and US8 act as antagonists of innate immunity by distinctively targeting TLR-signaling pathways. Nat Commun.

[94] Huang Y-W, Lin S-C, Wei S-C, Hu J-T, Chang H-Y (2013). Reduced Toll-Like Receptor 3 expression in chronic hepatitis B patients and its restoration by interferon therapy. Antiviral Therapy.

[95] Visvanathan K, Skinner NA, Thompson AJV, Riordan SM, Sozzi V (2007). Regulation of Toll-like receptor-2 expression in chronic hepatitis B by the precore protein. Hepatology.

[96] Delphin M, Faure-Dupuy S, Isorce N, Rivoire M, Salvetti A (2021). Inhibitory Effect of IL-1β on HBV and HDV replication and HBs antigen-dependent modulation of its secretion by macrophages. Viruses.

[97] Chiale C, Marchese AM, Robek MD (2021). Innate immunity and HBV persistence. Curr Opin Virol.

[98] Revie D, Salahuddin SZ (2014). Role of macrophages and monocytes in hepatitis C virus infections. World J Gastroenterol.

[99] Perdiguero B, Gómez CE, Di Pilato M, Sorzano COS, Delaloye J (2013). Deletion of the vaccinia virus gene A46R, encoding for an inhibitor of TLR signalling, is an effective approach to enhance the immunogenicity in mice of the HIV/AIDS vaccine candidate NYVAC-C. PLoS One.

[100] Stack J, Haga IR, Schröder M, Bartlett NW, Maloney G (2005). Vaccinia virus protein A46R targets multiple Toll-like-interleukin-1 receptor adaptors and contributes to virulence. J Exp Med.

[101] Langer S, Hammer C, Hopfensperger K, Klein L, Hotter D (2019). HIV-1 Vpu is a potent transcriptional suppressor of NF-κB-elicited antiviral immune responses. eLife.

[102] Ferreira EA, Clements JE, Veenhuis RT (2024). HIV-1 myeloid reservoirs — contributors to viral persistence and pathogenesis. *Curr HIV/AIDS Rep*.

[103] Veenhuis RT, Abreu CM, Costa PAG, Ferreira EA, Ratliff J (2023). Monocyte-derived macrophages contain persistent latent HIV reservoirs. Nat Microbiol.

[104] Stasakova J, Ferko B, Kittel C, Sereinig S, Romanova J (2005). Influenza A mutant viruses with altered NS1 protein function provoke caspase-1 activation in primary human macrophages, resulting in fast apoptosis and release of high levels of interleukins 1β and 18. J Gen Virol.

[105] Faure-Dupuy S, Depierre M, Fremont-Debaene Z, Herit F, Niedergang F (2024). Human rhinovirus 16 induces an ICAM-1-PKR-ATF2 axis to modulate macrophage functions. J Virol.

[106] Lundberg R, Melén K, Westenius V, Jiang M, Österlund P (2019). Zika virus non-structural protein NS5 inhibits the RIG-I pathway and interferon lambda 1 promoter activation by targeting IKK Epsilon. Viruses.

[107] Reynoso GV, Gordon DN, Kalia A, Aguilar CC, Malo CS (2023). Zika virus spreads through infection of lymph node-resident macrophages. Cell Rep.

[108] Konno Y, Kimura I, Uriu K, Fukushi M, Irie T (2020). SARS-CoV-2 ORF3b is a potent interferon antagonist whose activity is increased by a naturally occurring elongation variant. Cell Reports.

[109] Bai G, Zeng X, Zhang L, Wang Y, Ma B (2024). Computational investigation of the inhibitory interaction of IRF3 and SARS-CoV-2 accessory protein ORF3b. Biochem Biophys Res Commun.

[110] Kong K-F, Wang X, Anderson JF, Fikrig E, Montgomery RR (2008). West nile virus attenuates activation of primary human macrophages. Viral Immunology.

[111] Mazzon M, Jones M, Davidson A, Chain B, Jacobs M (2009). Dengue virus NS5 inhibits interferon-alpha signaling by blocking signal transducer and activator of transcription 2 phosphorylation. J Infect Dis.

[112] Zhang J, Zhao J, Xu S, Li J, He S (2018). Species-specific deamidation of cGAS by herpes simplex virus UL37 protein facilitates viral replication. Cell Host & Microbe.

[113] Ito S, Ansari P, Sakatsume M, Dickensheets H, Vazquez N (1999). Interleukin-10 inhibits expression of both interferon α– and interferon γ– induced genes by suppressing tyrosine phosphorylation of STAT1. Blood.

[114] Hwang E-H, Hur GH, Koo B-S, Oh H, Kim G (2022). Monocytes as suitable carriers for dissemination of dengue viral infection. Heliyon.

[115] Cooper GE, Pounce ZC, Wallington JC, Bastidas-Legarda LY, Nicholas B (2016). Viral inhibition of bacterial phagocytosis by human macrophages: redundant role of CD36. PLoS One.

[116] Jubrail J, Africano-Gomez K, Herit F, Mularski A, Bourdoncle P (2020). Arpin is critical for phagocytosis in macrophages and is targeted by human rhinovirus. EMBO Rep.

[117] Faure-Dupuy S, Jubrail J, Depierre M, Africano-Gomez K, Öberg L (2024). ARL5b inhibits human rhinovirus 16 propagation and impairs macrophage-mediated bacterial clearance. EMBO Rep.

[118] Faure-Dupuy S, Depierre M, Fremont-Debaene Z, Herit F, Niedergang F (2024). Human rhinovirus 16 induces an ICAM-1-PKR-ATF2 axis to modulate macrophage functions. J Virol.

[119] George SN, Garcha DS, Mackay AJ, Patel ARC, Singh R (2014). Human rhinovirus infection during naturally occurring COPD exacerbations. Eur Respir J.

[120] Jubrail J, Kurian N, Niedergang F (2017). Macrophage phagocytosis cracking the defect code in COPD. Biomed J.

[121] Gredmark S, Tilburgs T, Söderberg-Nauclér C (2004). Human cytomegalovirus inhibits cytokine-induced macrophage differentiation. J Virol.

[122] Baasch S, Giansanti P, Kolter J, Riedl A, Forde AJ (2021). Cytomegalovirus subverts macrophage identity. Cell.

[123] Brown EJ, Frazier WA (2001). Integrin-associated protein (CD47) and its ligands. Trends Cell Biol.

[124] Cong L, Sugden SM, Leclair P, Lim CJ, Pham TNQ (2021). HIV-1 Vpu promotes phagocytosis of infected CD4 ^+^ T cells by macrophages through downregulation of CD47. *mBio*.

[125] Lê-Bury G, Deschamps C, Dumas A, Niedergang F (2016). Phagosome migration and velocity measured in live primary human macrophages infected with HIV-1. J Vis Exp.

[126] Dumas A, Lê-Bury G, Marie-Anaïs F, Herit F, Mazzolini J (2015). The HIV-1 protein Vpr impairs phagosome maturation by controlling microtubule-dependent trafficking. J Cell Biol.

[127] Lê-Bury G, Niedergang F (2018). Defective phagocytic properties of HIV-infected macrophages: how might they be implicated in the development of invasive *Salmonella typhimurium*?. Front Immunol.

[128] Ghigo E, Kartenbeck J, Lien P, Pelkmans L, Capo C (2008). Ameobal pathogen mimivirus infects macrophages through phagocytosis. PLoS Pathog.

[129] Hung H-Y, Lai H-H, Lin H-C, Chen C-Y (2023). The impact of sofosbuvir/velpatasvir/voxilaprevir treatment on serum hyperglycemia in hepatitis C virus infections: a systematic review and meta-analysis. Annals of Medicine.

[130] Ansems K, Grundeis F, Dahms K, Mikolajewska A, Thieme V (2021). Remdesivir for the treatment of COVID-19. Cochrane Database Syst Rev.

[131] Riccardo BA, Gabriele S, Nunzia E, Isabella DF, Biagio P (2024). Casirivimab and imdevimab for pregnant women hospitalized for severe coronavirus disease 2019. Am J Perinatol.

[132] Märtson A-G, Sturkenboom MGG, Knoester M, van der Werf TS, Alffenaar J-WC (2022). Standard ganciclovir dosing results in slow decline of cytomegalovirus viral loads. J Antimicrob Chemother.

[133] Tate MD, Brooks AG, Reading PC (2011). Correlation between sialic acid expression and infection of murine macrophages by different strains of influenza virus. Microbes Infect.

[134] Wong ZX, Jones JE, Anderson GP, Gualano RC (2011). Oseltamivir treatment of mice before or after mild influenza infection reduced cellular and cytokine inflammation in the lung. Influenza Resp Viruses.

[135] Lai L, Hui C-K, Leung N, Lau GK (2006). Pegylated interferon alpha-2a (40 kDa) in the treatment of chronic hepatitis B. Int J Nanomedicine.

[136] Shepherd J, Jones J, Hartwell D, Davidson P, Price A (2007). Interferon alfa (pegylated and non-pegylated) and ribavirin for the treatment of mild chronic hepatitis C: a systematic review and economic evaluation. Health Technol Assess.

[137] Hunt PW, Shulman NS, Hayes TL, Dahl V, Somsouk M (2013). The immunologic effects of maraviroc intensification in treated HIV-infected individuals with incomplete CD4+ T-cell recovery: a randomized trial. Blood.

[138] Bohannon DG, Zablocki-Thomas LD, Leung ES, Dupont JK, Hattler JB (2024). CSF1R inhibition depletes brain macrophages and reduces brain virus burden in SIV-infected macaques. Brain.

[139] Schreiber A, Rodner F, Oberberg N, Anhlan D, Bletz S (2024). The host-targeted antiviral drug zapnometinib exhibits a high barrier to the development of SARS-CoV-2 resistance. Antiviral Res.

[140] Thom RE, D’Elia RV (2024). Future applications of host direct therapies for infectious disease treatment. Front Immunol.

[141] McCormick TS, Hejal RB, Leal LO, Ghannoum MA (2022). GM-CSF: orchestrating the pulmonary response to infection. Front Pharmacol.

[142] Lang FM, Lee KM-C, Teijaro JR, Becher B, Hamilton JA (2020). GM-CSF-based treatments in COVID-19: reconciling opposing therapeutic approaches. Nat Rev Immunol.

[143] Bosteels C, Van Damme KFA, De Leeuw E, Declercq J, Maes B (2022). Loss of GM-CSF-dependent instruction of alveolar macrophages in COVID-19 provides a rationale for inhaled GM-CSF treatment. Cell Rep Med.

[144] Lucifora J, Bonnin M, Aillot L, Fusil F, Maadadi S (2018). Direct antiviral properties of TLR ligands against HBV replication in immune-competent hepatocytes. Sci Rep.

[145] Bhat MF, Srdanović S, Sundberg L-R, Einarsdóttir HK, Marjomäki V (2024). Impact of HDAC inhibitors on macrophage polarization to enhance innate immunity against infections. Drug Discov Today.

[146] Locatelli M, Faure-Dupuy S (2023). Virus hijacking of host epigenetic machinery to impair immune response. J Virol.

[147] Feng Q, Su Z, Song S, Χu H, Zhang B (2016). Histone deacetylase inhibitors suppress RSV infection and alleviate virus-induced airway inflammation. Int J Mol Med.

[148] Xiao Q, Guo D, Chen S (2019). Application of CRISPR/Cas9-based gene editing in HIV-1/AIDS therapy. Front Cell Infect Microbiol.

[149] Hu W, Kaminski R, Yang F, Zhang Y, Cosentino L (2014). RNA-directed gene editing specifically eradicates latent and prevents new HIV-1 infection. Proc Natl Acad Sci USA.

[150] Wang Q, Liu S, Liu Z, Ke Z, Li C (2018). Genome scale screening identification of SaCas9/gRNAs for targeting HIV-1 provirus and suppression of HIV-1 infection. Virus Res.

[151] Wang Y, Galkin A, Shang X, Marin A, Jin S (2025). Rational design of flavivirus E protein vaccine optimizes immunogenicity and mitigates antibody dependent enhancement risk. Nat Commun.

[152] Yamanaka A, Rattanaamnuaychai P, Matsuda M, Suzuki R, Matsuura Y (2022). Engineered flavivirus vaccines control induction of crossreactive infection-enhancing and -neutralizing antibodies. Vaccine.

[153] Teo A, Tan HD, Loy T, Chia PY, Chua CLL (2023). Understanding antibody-dependent enhancement in dengue: are afucosylated IgG1s a concern?. PLoS Pathog.

